# Anti-Müllerian hormone and progesterone levels produced by granulosa cells are higher when derived from natural cycle IVF than from conventional gonadotropin-stimulated IVF

**DOI:** 10.1186/s12958-015-0017-0

**Published:** 2015-03-24

**Authors:** Zahraa Kollmann, Nick A Bersinger, Brett D McKinnon, Sophie Schneider, Michael D Mueller, Michael von Wolff

**Affiliations:** Department of Obstetrics and Gynecology, Inselspital Berne, Berne University Hospital, Effingerstrasse 102, 3010 Berne, Switzerland; Department of Clinical Research, University of Berne, Murtenstrasse 35, 3010 Berne, Switzerland

**Keywords:** Human granulosa cells, Natural cycle, Anti-Müllerian hormone, Follicle-stimulating hormone receptor, Progesterone, IVF

## Abstract

**Background:**

The study was designed to compare the effect of *in vitro* FSH stimulation on the hormone production and gene expression profile of granulosa cells (GCs) isolated from single naturally matured follicles obtained from natural cycle *in vitro* fertilization (NC-IVF) with granulosa cells obtained from conventional gonadotropin-stimulated IVF (c-IVF).

**Methods:**

Lutein granulosa cells from the dominant follicle were isolated and cultured in absence or presence of recombinant FSH. The cultures were run for 48 h and six days. Messenger RNA (mRNA) expressions of anti-Müllerian hormone (AMH) and FSH receptor were measured by quantitative polymerase chain reaction (qPCR). AMH protein and progesterone concentration (P4) in cultured supernatant were measured by ELISA and RIA.

**Results:**

Our results showed that the mRNA expression of AMH was significantly higher in GCs from NC- than from c-IVF on day 6 after treatment with FSH (1 IU/mL). The FSH stimulation increased the concentration of AMH in the culture supernatant of GCs from NC-IVF compared with cells from c-IVF. In the culture medium, the AMH level was correlated significantly and positively to progesterone concentration.

**Conclusions:**

Differences in the levels of AMH and progesterone released into the medium by cultured GC as well as in AMH gene expression were observed between GCs obtained under natural and stimulated IVF protocols. The results suggest that artificial gonadotropin stimulation may have an effect on the intra-follicular metabolism. A significant positive correlation between AMH and progesterone may suggest progesterone as a factor influencing AMH secretion.

## Background

Follicle growth is a complex process involving a functioning and bidirectional communication between each oocyte and its surrounding somatic cell compartments [1-3]. At the antral stage of follicular growth most follicles undergo atresia, although due to gonadotropin stimulation, some will enter the pre-ovulatory stage [4-6]. However, the exact mechanism of follicle selection and primordial follicle activation is not yet completely understood.

The follicle-stimulating hormone (FSH) and its receptor (FSHR) play an essential role in recruitment and follicle growth [7,8]. In humans the dominant follicle selection may depend on differential FSH sensitivity amongst a growing cohort of small antral follicles. Various growth factors related to the TGFβ superfamily contribute to this selection process by interacting with gonadotropin-induced signals [9-11]. Anti-Müllerian hormone (AMH) is one such growth factor that is produced by granulosa cells (GCs) and present in small and large pre-antral follicles, with maximal levels observed in secondary, preantral and small antral follicles ≤ 4 mm [7]. AMH may play an important role in primordial follicle selection and cyclic growing follicle recruitment [12]. Moreover, AMH might regulate the selection of the dominant follicle through the inhibitory effects of AMH on the initial recruitment of primary follicles from the resting primordial follicle pool [13-15] and through the regulation of FSH, sensitivity in the human ovary [16].

In addition, recent research has suggested a relationship exists between follicular fluid AMH concentrations of the pre-ovulatory follicle and the occurrence of a clinical pregnancy. Embryo implantation rates were higher when oocytes were obtained from follicles with high AMH concentrations in the follicular fluid, but not in the serum on cycle day 3 and on the day of oocyte pickup [17,18]. Furthermore, it has been shown that follicular AMH concentrations were approximately three-fold higher in NC-IVF follicles than in c-IVF-follicles at the time of ovum pickup [19]. In addition, previous studies have shown that the implantation potential of oocytes is higher in NC-IVF [20,21] compared with c-IVF [22], and it can be expected that the NC-IVF follicles would closely represent physiologically normal follicles. Thus it can be assumed that the artificial stimulation of follicle growth that occurs in c-IVF treatment has certain effects on the follicles. In the context of a project studying the difference and clinical consequences between natural and stimulated IVF treatment cycles with different approaches, we have decided to set up a series of *in vitro* experiments to assess the endocrine function of GCs derived from both NC-IVF and c-IVF protocols. This was done by measuring the AMH production and secretion and the progesterone (P4) secretion by cultured granulosa cells both with and without FSH stimulation.

## Methods

### Study population and treatment cycles

The study was approved by the local ethical committee before commencement (reference no. 12–023, Inselspital Berne, Teaching and Research Management, IRB Internal Review Board, approved 11 October 2012) and patients' approval was given by written consent.

GCs were collected at oocyte retrieval from 41 women (mean age 36.5 +/− 3.8 (SD), range 28–42) and with regular cycles who had been referred to our clinic for infertility treatment between October 2012 and August 2013. The study population was divided into two groups according to the selected treatment protocol. (A) Patients (n = 21) undergoing NC-IVF without medical intervention except for the administration of hCG (Pregnyl; MSD, Switzerland) 36 h before follicle aspiration and (B) patients (n = 20) undergoing a stimulation cycle (c-IVF) with GnRH antagonist (Orgalutran, Ganirelix 0,25 mg, MSD, Switzerland) protocol and using highly purified hMG (Menopur, Menotropin hMG, Ferring Pharmaceuticals, Baar, Switzerland) for 8–10 days and daily doses of 150–300 IU of hMG, followed by hCG (Pregnyl). Transvaginal ultrasound guided oocyte retrieval was performed 36 h later. In c-IVF, granulosa cells were collected from the first aspirated matured (MII) large follicle (LF; > = 18 mm). The causes of infertility were mainly male or idiopathic in both groups; the individual numbers are given in Table 1.

**Table 1:** **Study population and ovarian cycle characteristics**

**Variables**	**Group A**	**Group B**	**P**
	**NC-IVF**	**cIVF**	
Patients	21	20	ns
Age, mean +/− SEM (yrs)	35.4 +/− 0.8	37.6 +/− 0.8	ns
*Range*	28-41	31-42	
Etiology of infertility (n/Total) *Male factor*	7/21	8/20	
*Tubal factor*	4/21	2/20	
*Endometriosis*	4/21	2/20	
*Idiopathic*	6/21	8/20	
AMH (pmol/L)	14.2 +/− 3.7	22.4 +/− 5.1	0.1430
Number of oocytes retrieved	1.0 +/− 0.0	6.9 +/− 1.2	<0.0001***
Follicle Diameter (mm)	18.2 +/− 0.4	18.5 +/− 0.4	ns

*Data other than P values are mean +/− SEM.*

### Collection and culture of granulosa cells

Immediately after oocyte retrieval and the isolation of the cumulus oophorus complex, granulosa cells were isolated from the follicular fluid and flushing medium [23]. Only the leading follicle was analysed in stimulated cycles without pooling. The total aspirate volume was collected in a 15-ml polystyrene test tube (BD Falcon) and centrifuged at 440 × *g* for 10 min. The GCs containing pellets were clearly visible and were separated, avoiding aggregates with red blood cells as described elsewhere [24], with the exception of the density gradient, which had to be omitted due to the small number of GCs present in single follicles. The Pellets were suspended in 0.4 mL freezing medium (Iscove’s modified Dulbecco medium, IMDM, Gibco-Ivitrogen, Paisley, Scotland) without phenol red and with DMSO (10% v/v) and stored at −80°C until used in the culture experiment. To ensure that sufficient GC populations had been obtained in the flushing procedure, a direct smear slide was made from the pellet for each sample. The smear slides were prepared by cyto-centrifugation, fixed immediately with Cytostat 400® spray (Simat AG, Glattbrugg, Switzerland) and stained with Papanicolau (PAP) reagent [25].

For the culture experiment the isolated GCs were thawed at 37°C and washed twice in culture medium (IMDM containing foetal bovine serum (10% v/v), penicillin, streptomycin and Fungizone (all from Gibco-Invitrogen) and without phenol red. Cells were counted with Trypan blue for evaluation of cell concentration and viability, then seeded at 10,000 live cells/cm^2^ into 48-well plates and maintained in complete medium with foetal bovine serum and the antibiotic/antimycotic reagent. After 24 h of incubation in 1 ml total volume, the supernatants were then aspirated and medium containing recombinant FSH (Gonal-F®, 0.1 IU/mL or 1.0 IU/mL, Merck Serono S.A., Geneva, Switzerland) was added. One well from each sample was cultured as a control in the absence of FSH. The cultures were run for 48 h and six days. For the measurement of progesterone and AMH protein concentrations, 0.5 ml of supernatant was removed from each well after 48 h and the remaining volume on day 6 at conclusion of the culture. Supernatants were centrifuged at 1000 × *g* for 5 min. Progesterone (P4) was quantified using a commercially available radio-immunoassay (“Coat-a-Count”) from DPC, Los Angeles, USA. The 3-h incubation protocol at room temperature was used. For P4 determinations the introduced supernatant volume had to be diluted 1:5 in the zero standard as a carrier. The intra-assay coefficient of variance at the relevant P4 concentrations was between 3.6 and 4.9%. AMH was determined manually with commercially available specific microplate Enzyme-Linked Immunosorbent Assay (ELISA). The assay for AMH was obtained from Cusabio (China). The functional sensitivity of the assay was 0.375 ng/mL, and the intra- and inter-assay coefficients of variance were reported to be 15% each. Culture supernatants were diluted 1:2 in 0.9% NaCl prior to assay and the protocol of the manufacturer was followed. The hormone concentrations were expressed after normalisation by dividing their concentration by the cell number.

### Immunofluorescence staining of granulosa cells for FSH receptor

Granulosa cells were identified by the expression of the FSH receptor (FSHR). They were plated on cover slips at 10,000 live cells/cm^2^ into 48-well plates. After six days of culture, cells were fixed in paraformaldehyde (4% w/v, Grogg Chemie, Stettlen, Switzerland) for 10 min. and rinsed five times in PBS containing Tween-20 (0.1% v/v, PBST). The slides were incubated with the primary antibody against FSH receptor (monoclonal mouse anti-human FSHR, R&D Systems, England, dilution 1:50), 5% normal goat serum (NGS), 0.5% casein, in PBS for 90 min. at room temperature After three washes in PBST, the slides were incubated with the second antibody, chicken anti-mouse IgG (H + L) labelled with Alexa® Fluor 488 (Invitrogen, USA, dilution 1:200) for 1 h at room temperature. Then, the slides were washed another three times in PBST. Nuclei were stained with 4’,6-diamidinmo-2-phenylindole (DAPI, sigma) at a final concentration of 0.5 μg/mL for 10 min. and washed three times for 10 min. in PBST before mounting in UltraCruz™ Mounting Medium sc-24941 (Santa Cruz Biotechnology Inc.). Fluorescence images were captured under an Axiovert 40 CFL with AxioCam MRm.

### Determination of gene expression

Samples with a useful quantity of RNA were extracted from 14 single follicles (NC-IVF) out of n = 21. Another 14 samples were collected from the c-IVF group with a total number of 20 samples. Extraction of RNA was achieved by using the Rneasy*®* Plus Micro Kit (Qiagen, Hilden, Germany) according to the manufacturer’s protocol. RNA quantity and purity were determined with a Nanodrop*®* Spectrophotometer (Wilmington, USA). Reverse transcription (RT) was performed with the Quantitech Reverse Transcription kit (Qiagen, Hilden, Germany) with a concentration of 0.15 ng/μL RNA in 20 μL. A pre-amplification of cDNA was performed using TaqMan® PreAmp Master Mix Kit (AB, Applied Biosystems, CA, USA) for 14 cycles, as recommended by the manufacturer. The subsequent cDNA was used for quantitative PCR (qPCR) in 20 μL reaction volumes using the TaqMan® Gene Expression assays (Applied Biosystems Europe, Zug, Switzerland), in an Applied Biosystems 7900 HT Fast Real Time PCR System. The following primers/probes were used: AMH (NM_000479, Hs01006984); FSHR (NM_000145, Hs00174865); with GAPDH (NM_002046, Hs03929097) and ACTB (NM_001101, Hs00242273) as reference genes.

The relative gene expression was calculated with the qBASEplus software (Biogazelle, Zwijnaarde, Belgium) using a method based on the ΔΔCt method that incorporates the use of multiple reference genes.

### Statistical analysis

All statistical analyses were performed using GraphPad Prism. The non-parametric Mann–Whitney U-test was performed to compare the expression of mRNA between the GCs from the cIVF group and cells from NC-IVF. For analysis of variance, the two-way ANOVA test was used to compare the effect of IVF method and the effect of FSH treatment. The Spearman Rank Correlation Analysis was applied to determine whether there was a significant relationship between progesterone and AMH concentrations in the culture medium. The significance level was set at p < 0.05. All data are expressed as the mean +/− SEM.

## Results

The clinical parameters in NC-IVF and c-IVF patients are summarized in Table 1. There were no differences in patient age, AMH level in serum or follicle diameter between treatment groups.

### Cell morphology

**Figure 1:**
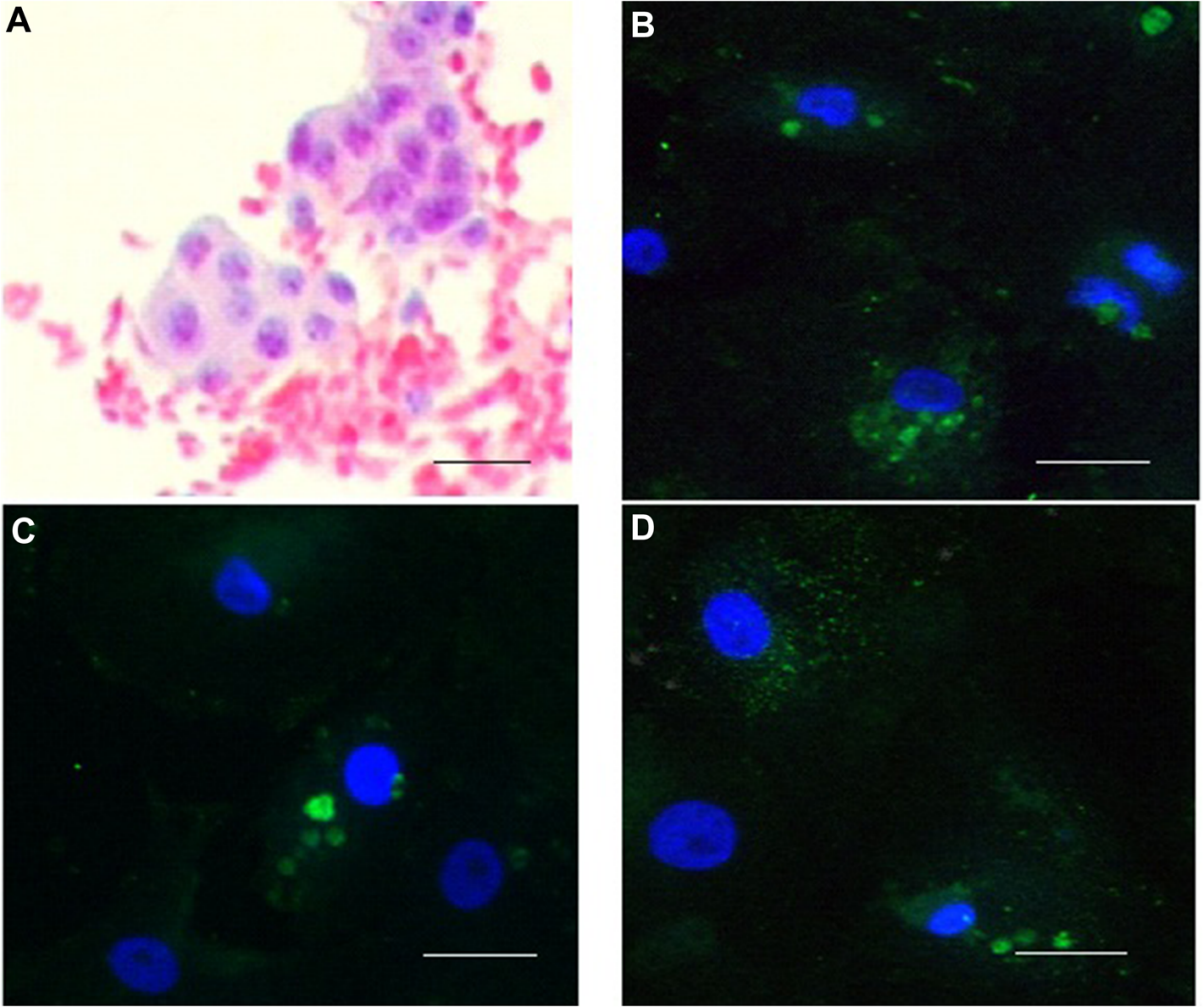
**Staining for Granulosa cells and Immunofluorescence staining for FSH receptor. (A)** Aggregated granulosa cells stained with Papanicolaou (20x magnification). Bar = 20 μm. Localisation of FSH receptor (FSHR) in human granulosa cells (GC) after six days in culture, Immunofluorescence FSHR staining with monoclonal mouse anti-human FSHR (primary), chicken anti-mouse (Alexa, secondary), counterstained with DAPI for nuclei. **(B)** GCs in control culture (without FSH) showing cytoplasmic vesicles; **(C)** GC culture stimulated with FSH 0.1 IU/mL showing outgrowth with fibroblast-like morphology; **(D)** GC culture stimulated with FSH 1 IU/mL showing an intracellular signal for FSHR which could indicate *de novo* production (40x magnification). Bar = 40 μm.

In the PAP staining method nuclei appear as homogeneously dark blue chromatin whereas the cytoplasm appears as a pale eosinophilic coloration (Figure 1A).

Human granulosa cells in culture attached within 24 h, the red blood cells were visible in the initial culture, but did not remain after gentle shaking and a change of the media after 24 h of culture. The FSHR in the GCs were identified using immunostaining on day 6. Cells cultured and treated with FSH (0.1 IU/ml, 1 IU/mL) have shown a fibroblast-like morphology and an intracellular signal for FSHR (Figure 1C, D).

### Effect of FSH on the expression of follicle stimulating receptor and of AMH mRNA

**Figure 2:**
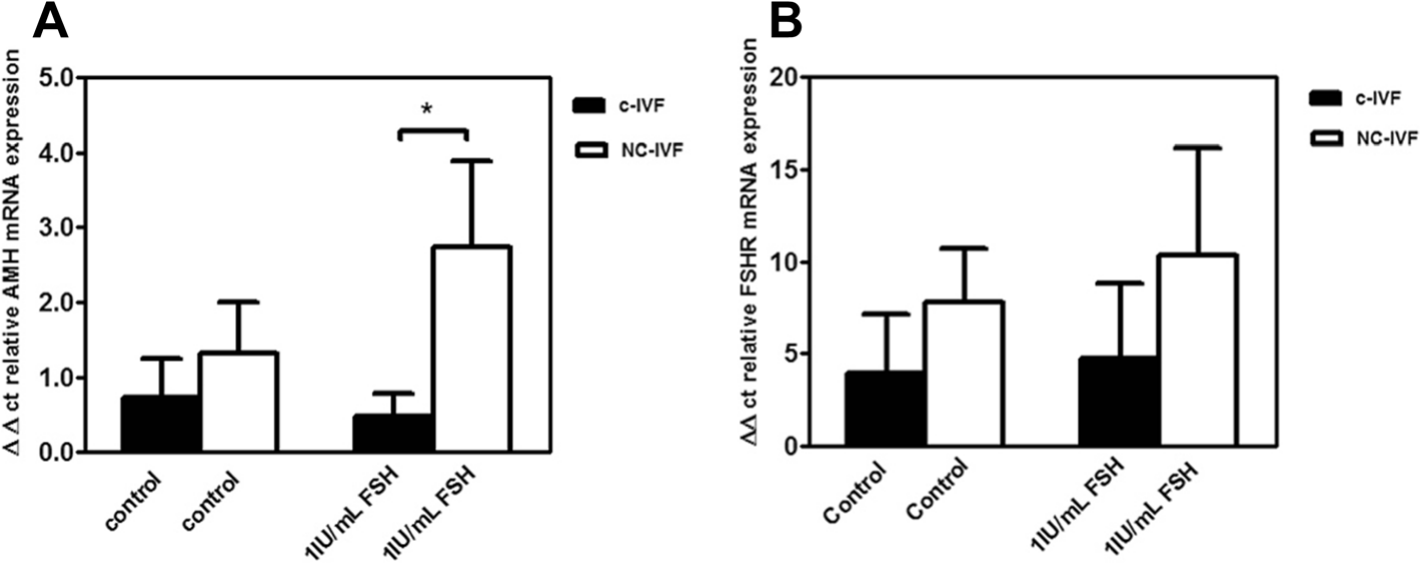
**Effect of FSH on the expression of FSHR- and of AMH- mRNA in GCs from NC-IVF and c-IVF.** Gene expression of AMH mRNA **(panel A)** and FSHR mRNA **(panel B)** levels by qPCR in the granulosa cell lysate after six days of culture in 28 GC preparations (NC-IVF, N = 14, open bars; c-IVF, N = 14, closed bars).

NC-IVF GCs stimulated for six days with 1 IU/mL FSH had a significantly higher AMH mRNA expression compared with c-IVF GCs (3.41 +/− 1.55, n = 14 *vs.* 0.053 +/− 0.43, n = 14; *p* = 0.0344) (Figure 2A). There was no significant difference in the expression of FSHR mRNA (4.76 +/− 4.06, n = 14 *vs.* 10.34 +/− 5.82, n = 14) in cells obtained from NC or c-IVF after 1 IU/mL FSH (Figure 2B).

### Effect of FSH on AMH protein levels in the culture media of GCs

**Figure 3:**
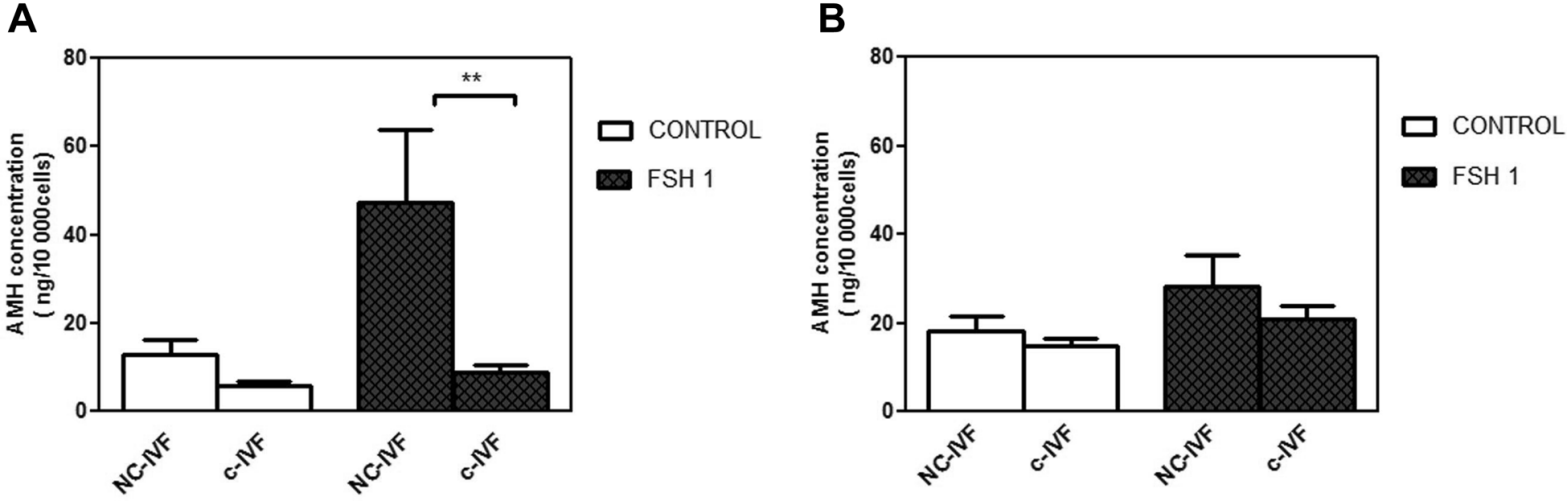
**Effect of FSH on AMH concentrations in the culture media of GCs from NC-IVF and c-IVF.** Comparison of AMH concentrations in the culture medium of granulosa cells (GCs) isolated from conventionally stimulated IVF (c-IVF, N = 20) and from natural cycle IVF (NC-IVF, N = 21), cultured in absence (control, open bars) and presence of FSH (1.0 IU/mL, closed bars). Incubation in culture was 48 h **(Graph A)** and 6 days **(Graph B)**. Data are presented as the mean +/− SEM.

The mean AMH concentration in the conditioned medium from GCs derived from NC-IVF in the absence of FSH showed no significant difference from the mean AMH concentration from the c-IVF GCs after 48 h culture (12.9 +/− 1.2, n = 21 *vs*. 5.8 +/− 2.5 ng/10,000 cells, n = 20). While after 1 IU/mL FSH treatment at 48 h there was a significant difference between the NC-IVF and c-IVF GCs (47.4 +/− 4.2, n = 21 *vs.* 8.7 +/− 3.0 ng/10,000 cells, n = 20). FSH (1 IU/mL) increased the AMH concentration in the culture medium of GCs from NC-IVF significantly (*p* = 0.0201) when compared with control group without FSH. Moreover, the level of AMH was significant higher in treated GCs from NC-IVF with FSH (1 IU/mL) when compared with treated GCs from c-IVF (*p* = 0.0046) (Figure 3A). After six days of culture, both the unstimulated (control groups) and 1 IU/mL FSH-stimulated GCs groups did not exhibit a significant difference between the NC-IVF and the c-IVF, see (Figure 3B). In the control group from NC-IVF and c-IVF, the concentration of AMH was (18.1 +/− 2.9, n = 21 *vs.* 14.8 +/− 1.8 ng/10,000 cells, n = 20). In the FSH-stimulated group, the concentration of AMH in NC-IVF decreased after six days, but it remained slightly higher than in c-IVF (28.1 +/− 8.0, n = 21 *vs*. 20.9 +/− 4.0 ng/10,000 cells, n = 20).

### Effect of FSH on progesterone concentration in the culture media of GCs

**Figure 4:**
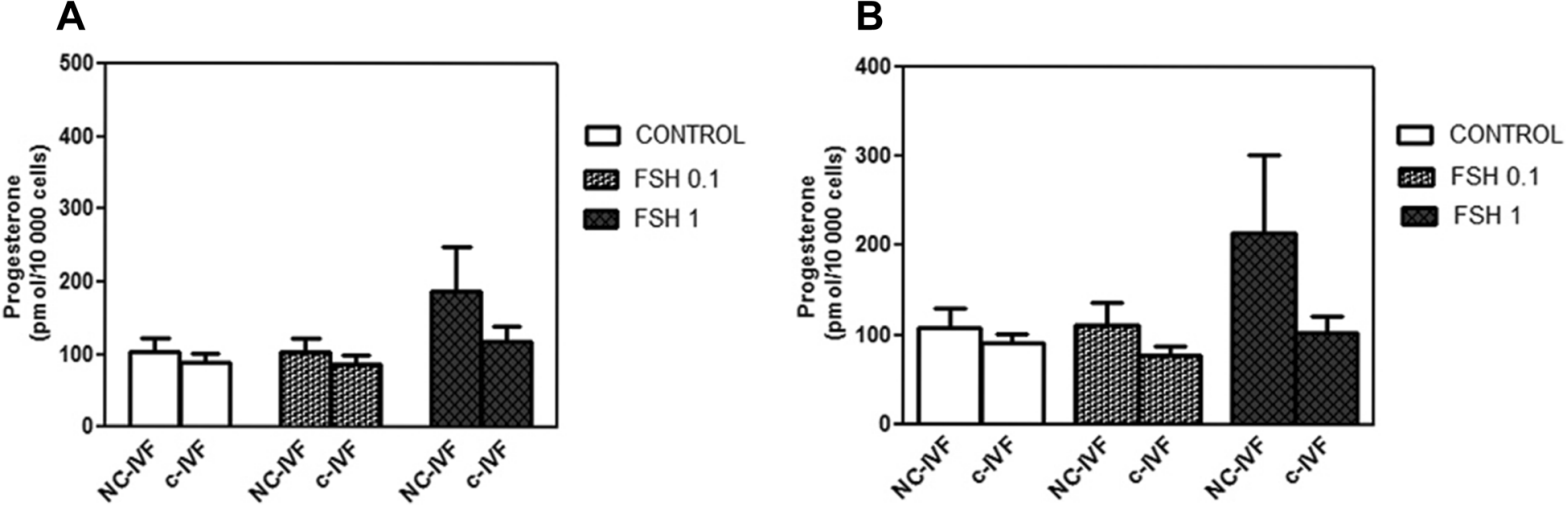
**Effect of FSH on progesterone concentration in the culture media of GCs from NC-IVF and c-IVF.** Concentration of progesterone (P4) in the culture medium of granulosa cells (GCs) isolated from conventionally stimulated (c-IVF, N = 20) and from natural cycle IVF (NC-IVF, N = 21), cultured in absence (control) and presence of FSH (0.1 IU/mL, 1.0 IU/mL). Incubation in culture was 48 h **(Graph A)** and 6 days **(Graph B)**. Data are presented as the mean +/− SEM.

A higher but non-significant increase in P4 was observed in the GCs from NC-IVF compared with c-IVF (188.0 +/− 60.1, n = 21 *vs.* 119.0 +/− 20.7 pmol/10,000 cells, n = 20) after a 48-h treatment with 1 IU/mL FSH (Figure 4A). On day 6, P4 showed a similar pattern of increasing, but it was not significant in the culture medium from NC-IVF GCs compared with c-IVF GCs in the presence of 1 IU/mL FSH (201.0 +/− 93.3, n = 21 *vs.* 101.09 +/− 19.3 pmol/10,000 cells, n = 20) (Figure 4B). A significant positive correlation after six days of treatment with 1 IU/mL FSH between Progesterone and AMH concentrations from both NC-IVF GCs (r = 0.89, *p* < 0.0001) and from c-IVF GCs (r = 0.64, *p* = 0.014) was observed.

## Discussion

The objective of this study was to compare the GCs obtained from c-IVF and NC-IVF by analyzing the production of both progesterone and AMH and their subsequent responsiveness to FSH exposure *in vitro*. The results indicate that both groups of GCs retain FSHR, the expression of which can be further induced by FSH treatment. The NC-IVF GCs, however, produced significantly more AMH mRNA and secreted significantly more AMH upon FSH exposure. In addition, a six-day FSH treatment of both groups resulted in the secretion of more P4 from the NC-IVF cells compared with the c-IVF cells. The results of this study therefore suggest that GCs from NC-IVF retain a higher sensitivity to FSH compared with GCs from c-IVF. It is therefore possible that c-IVF stimulation may result in GCs reaching their peak progesterone production capabilities during the stimulatory cycle.

A major finding of this study is the increased AMH mRNA expression in GCs from NC-IVF after a six-day FSH (1 IU/mL) treatment. Previous studies on the relationship between FSH and AMH in GC cultures have been inconsistent. Taieb et al. [26] showed that both FSH and cAMP up-regulated AMH mRNA expression in human luteinized GCs, whereas Pellatt et al. [27] did not detect any effect of FSH on AMH production by GCs derived from normal ovaries, but in GCs from women with polycystic ovaries FSH stimulation decreased significantly AMH levels. A further study also reported a FSH-stimulated AMH increase in GCs from both normo-ovulatory and oligo/anovulatory PCOS women [28]. The observations from this latter group support our results and suggest that elevated AMH secretion in cultured GCs from NC-IVF would not depend on the previous production of the hormone in small antral follicles.

GCs from NC-IVF also showed a significant increase in *in vitro* secretion of AMH after 48 h exposure to 1 IU/mL FSH. After six days this increase in AMH secretion from NC-IVF compared with c-IVF was still present, however the higher variation between the different culture preparations may have hindered the analysis of statistical significance. Our results from both protein (Figure 3) and gene expression (Figure 2A) for AMH show the same trend as previously reported for the follicular fluid concentrations of the same hormone [19] although statistical significance was not reached in the absence of FSH stimulation *in vitro*. The expression of the FSH receptor and the effects of FSH stimulation vary throughout the different stage of GC differentiation [29]. The mechanism of how gonadotropins affect AMH secretion in GC is not clear, although it is possible that the follicular and the luteal phases have differing dynamics [30,31].

Another finding which we consider important was that after a six-day culture, P4 concentrations were increased in NC-IVF compared with the c-IVF after 1 IU/mL FSH and that AMH secretion was correlated with P4 concentrations in the both groups but especially strongly in the NC-IVF group. The long incubation time has been initially chosen in order to reach a higher sensitivity for both the absolute hormone levels in the supernatant and for the difference in these levels between the different hormone treatments. A shorter, potentially more physiologically relevant incubation period would have been difficult to control for technical reasons.

P4 is the major steroid synthesized by luteinized GCs [32], and it is therefore possible that the luteinization of GCs changes the follicular capacity to produce AMH. Previous evidence shows that the level of P4 declines during final follicular maturation [33,34], and the degree of this maturation and luteinization may influence AMH production by the GCs [30]. It has also been shown that the serum and follicular levels of AMH decline immediately after hCG administration but rise again four days later [30]. Similarly, it has been reported that in natural IVF cycles an alteration in the hormonal milieu after the LH surge increases serum AMH levels on the day of follicle aspiration, which reflects early pre-ovulatory changes after hCG administration [31]. Therefore, these results suggest that c-IVF results in lower concentrations of AMH secreted from GCs. A number of reasons for this reduced production and secretion are possible: First, c-IVF is characterized by the growth of several follicles following multiple follicle recruitment during the initial stimulation step compared with NC-IVF. It can be speculated that the non-physiological recruitment of multiple follicles results in intrinsic abnormalities in the ovarian follicles themselves and in relation to the numbers of recruited and aspirated follicles. This is suggested by several studies that investigated follicular and serum AMH levels after stimulation with menopausal and recombinant gonadotropins [35-37]. Second, an exogenous gonadotropin administration during the follicular phase would correlate with a decrease of AMH secretion. A study which compared the changes of serum AMH levels between spontaneous cycles and FSH treated cycles during the follicular phase did not find a change in AMH plasma levels in the spontaneous cycles, but did find a change in AMH plasma levels in the FSH treated cycles [38].

A minor limitation of the current study is the use of human lutein GCs. This is not the best model to study the regulation of AMH by gonadotropins because these cells have been exposed to exogenous hCG administration. Because of this exposure to hCG administration, *in vitro* studies do not perfectly reflect the *in vivo* situation during follicular maturation. However, progesterone production is an accepted marker for GC responsiveness to FSH stimulation.

## Conclusions

Our study has shown that luteinized granulosa cells obtained on the day of oocyte retrieval after either c-IVF or NC-IVF react differently to FSH stimulation *in vitro*. GCs from NC-IVF retain their physiological capacity to respond to FSH stimulation by increasing their production and secretion of AMH and progesterone. Moreover, the reduced AMH production of GCs obtained from c-IVF follicles suggests that for future research, treatment with exogenous gonadotropins might force the production of oocytes even from non-competent follicles. If an adaption of the stimulation protocols in c-IVF could improve the intrafollicular metabolism and thereby increases the oocyte quality, remains to be evaluated.
